# ModuleBlast: identifying activated sub-networks within and across species

**DOI:** 10.1093/nar/gku1224

**Published:** 2014-11-26

**Authors:** Guy E. Zinman, Shoshana Naiman, Dawn M. O'Dee, Nishant Kumar, Gerard J. Nau, Haim Y. Cohen, Ziv Bar-Joseph

**Affiliations:** 1 Lane Center for Computational Biology, School of Computer Science, Carnegie Mellon University, Pittsburgh, PA 15213, USA; 2 The Mina & Everard Goodman Faculty of Life Sciences, Bar-Ilan University, Ramat-Gan 52900, Israel; 3 Department of Microbiology and Molecular Genetics, School of Medicine, University of Pittsburgh, Pittsburgh, PA 15216, USA

## Abstract

Identifying conserved and divergent response patterns in gene networks is becoming increasingly important. A common approach is integrating expression information with gene association networks in order to find groups of connected genes that are activated or repressed. In many cases, researchers are also interested in comparisons across species (or conditions). Finding an active sub-network is a hard problem and applying it across species requires further considerations (e.g. orthology information, expression data and networks from different sources). To address these challenges we devised ModuleBlast, which uses both expression and network topology to search for highly relevant sub-networks. We have applied ModuleBlast to expression and interaction data from mouse, macaque and human to study immune response and aging. The immune response analysis identified several relevant modules, consistent with recent findings on apoptosis and NFκB activation following infection. Temporal analysis of these data revealed cascades of modules that are dynamically activated within and across species. We have experimentally validated some of the novel hypotheses resulting from the analysis of the ModuleBlast results leading to new insights into the mechanisms used by a key mammalian aging protein.

## INTRODUCTION

Several studies rely on gene expression profiling (either using microarrays or RNA-Seq) to identify genes that are differentially expressed (DE) between treatment and control or to find genes that are involved in a specific condition. While such studies led to useful results, proteins usually operate in complexes or cascades and are often post transcriptionally regulated, so in many cases important genes may be missed when only using expression data. Interaction data are useful for identifying such genes (1) and an increasing number of studies attempted to integrate static gene and protein interaction data with dynamic expression data in order to find ‘active sub-networks’. Such sub-networks are connected regions within the global interaction network that contain several DE genes. Identifying such active sub-network allowed researchers to generate concrete testable hypotheses regarding the regulatory processes that underlie the observed changes in gene expression (1–7) Many researchers refer to active sub-networks as ‘modules’ underscoring the ability of these sub-networks to capture coherent functionality. In this manuscript we will use the terms ‘sub-networks’ and ‘modules’ interchangeably.

Several studies have utilized cross species expression data for studying the same condition in multiple species (8–10). Such analyses highlight the similarities and differences in key mechanisms between the species, improving our biological understanding both from an evolutionary point of view (11) and for the activity under specific conditions (12). These two types of analyses (sub-networks and cross species comparisons) have primarily remained separate with researchers either using one or the other in each study. While the active sub-networks approach can be an excellent tool for analyzing expression pattern differences between species and tracking the origins of these differences, to do so we need to overcome several challenges. These include orthology assignments, comparison of expression patterns across platforms and species and differences in the association networks.

Optimally finding active sub-networks or modules in a general graph was shown to be Non-deterministic Polynomial-time (NP) hard (1). Several heuristic and approximation algorithms have been proposed to identify such modules including methods that use pre-defined groups (13,14), simulated annealing (1,2,15), greedy search approaches (3,16–18), optimization algorithms (7,19) and methods based on graph theory (20). See Supplementary Table S1 and (21) for further details. In practice, greedy search approaches were shown to obtain good results with significantly shorter runtime compared to other approaches (16). We are aware of only one attempt to combine active sub-network discovery with cross species analysis (22). While NeXus performs well for some data, it does not allow simultaneous analysis of both conservation and divergence. In addition, as we show in the Results section, NeXus identifies several hundreds of highly overlapping modules which may make it hard for a biologist to focus on a few modules for follow up analysis and experiments. We have thus developed a novel method, ModuleBlast, which addresses the need for an integrated analysis of network data across species allowing for the identification of both conserved and divergent sub-networks. Another important aspect missing from prior work on cross species sub-network analysis that we address with our new method is using temporal data. ModuleBlast can link modules over different time points to identify causal effects leading to expression changes. Our method is based on integrating expression and interaction data from two or more species and searching for active modules using the absolute activation in all species. Module search expands highly activated seeds into modules as long as the overall activation of the modules is maximized. We have also built a web application that supports both single species and cross species module searches.

We used ModuleBlast to study the temporal response of murine and macaque macrophages to *Francisella tularensis*, a gram-negative bacterium that is highly virulent in humans (23) and to perform cross species studies of aging. The resulting modules provide an overview of the similarities and differences in the response across species. These modules were used to generate new hypotheses regarding cellular responses to infection and the role of a key aging protein, SIRT6, a number of which were validated in follow-up experimental studies.

## MATERIALS AND METHODS

We first discuss our method for generating a cross species network that can be used to identify both similarities and differences between species. Once we have networks that integrate information across species, we use a novel target function that takes into account both activity and connectivity to search for active sub-networks. We present a greedy search method for finding modules (sub-networks) that maximize the target function and discuss how such modules can be connected in time when using time series expression data to identify the progression of information within cells.

### Generating cross species gene association networks

We assembled gene association networks using various genomic data types including protein–protein interactions and genetic interactions from BioGRID (24), version 2.0.63. To combine multiple species we created networks in which nodes correspond to entire orthogroups containing gene orthologs from all the species that are part of the analysis based on Inparanoid (25) orthogroup definitions. We are interested in finding sub-networks with high activation, regardless of the species, therefore the node score is set to be the absolute of the most extreme value in the orthogroups (Supplementary Figure S1). Edges between nodes in our network include interactions connecting any of the genes in the two orthogroups. Interactions can be weighted according to the confidence in the interaction if one is provided (e.g. the log likelihood score) and summed across various data types and species. As these weights are comparable across species, it is possible to merge association networks from several species into a single association network. Edges that have evidence in more than one species have a higher weight indicating the increased confidence in seeing this edge across species. As in this study, we used for both species interactions only from BioGRID, which does not provide confidence measurement for the interactions, the edge score would be the number of evidences observed for an interaction in both species combined. Integrating data from several species into a single gene association network allows us to overcome some of these issues related to missing data of less studied species. We note that current interaction data, especially of higher organisms including mammals, are incomplete, and in this study human interactions contributed ∼90% of the edges. However, over 50% of the mouse interactions were also found in human. Our final joint network contained 6188 nodes and 21 655 unique edges. See Supplementary Data Set DS1 for species-specific details.

### Immune response expression data

Mouse and macaque expression data for the Schu S4 immune response analysis were downloaded from Array Express; accession number: E-MTAB-427. The data are from Zinman *et al.* (9). The data set contains a time series expression following the infection of Alveolar macrophages (AM) from mouse and macaque with the Schu S4 bacteria. Cells were harvested at 0, 1, 2, 6, 12 and 24 h post infection and were hybridized to Agilent arrays. Standard arrays (G4122F) were used for mouse samples, whereas custom arrays were used for *Macaca mulatta* using sequence data downloaded from the NCBI database.

### Aging expression data

The analysis of SIRT6 expression in mouse and human cells is based on data from (32). Following tumor necrosis factor alpha (TNF-α) treatment, genes were profiled in mouse embryonic fibroblast cells (MEF) extracted from a knockout SIRT6 mouse and compared to cells from a control wild-type mouse (GSE13207). A similar experimental setup was used by the same lab to compare the expression of human HeLa cell line treated with a short hairpin RNA against SIRT6 (shSIRT6 knockdown) with the expression in control pSR HeLa cells (GSE13206) (26).

### Node/edge score calculation

Node scores quantify the differential expression of treatment and control data for each gene (node) assuming Gaussian distribution for the control experiments. For the *F. tularensis* response analysis, we averaged the values of six time points following infection for both mouse and macaque as the treatment experiment response and compared these (in each species) to the average of the times series of mock values (see (9) for details). For the aging analysis we averaged the log fold change in time points 6 h and 24 h after treatment of TNF and shRNA in mouse and human, respectively.

Edges were derived from BioGRID (24), version 2.0.63 for mouse and human. The macaque network was based on the human interaction network by matching protein names. A new association network for the joint species analysis was created based on the union of all individual species edges that connect genes in any two orthogroups. Since BioGRID does not provide confidence measurement for the interactions, we defined the score of each edge by counting the number of sources supporting each interaction in the species-specific network, thus edges present in more than one species would have a higher weight in the joint network.

### Scoring sub-networks

Given a connected sub-network (module *j*), early studies (1) scored it by summing the node scores (which were assumed to be drawn from a standard normal distribution) with respect to background distribution:

(1) \begin{equation*} S_j = \frac{1}{{\sigma _Z }}\frac{{\sum\nolimits_i {Z_i - \beta _Z \mu _Z } }}{{\sqrt M _{} }}, \end{equation*}

where *Z_i_* is a node score for a node in module *j* based on the absolute of the most extreme value of the orthogroup and was shown to follow a normal distribution with parameters (*μ_Z_*,*σ_Z_*). The subscript *Z* refers to parameters pertaining to nodes. *M* is the number of nodes in sub-network *j*. *β_Z_* is an empirical parameter designed to produce fewer nodes with a positive score, hence creating smaller sub-networks (27). By subtracting *β_Z_ μ_Z_*, from each node score we obtain a sum over normal standard variables (*S_j_*), which is a normally distributed random variable with mean $\frac{\mu }{M}$ and standard deviation $\frac{\sigma }{{\sqrt M }}$. The advantage of such a scoring system is that it allows comparing sub-networks of different sizes (16). While node scores are useful, they can only identify transcriptionally regulated genes. In addition to node scores, our method also takes into account the strength of the interactions between genes in the modules. Note that by using such interactions, we may be able to include in the modules genes that are not DE but that are connected to many other DE genes. If such genes are indeed involved in similar pathways and functions as the DE genes, while not being DE themselves, they are likely post-transcriptionally regulated and may impact the expression of their neighbors in the network. We thus extended the node score objective function leading to a target function that is a weighted sum of two components; nodes and edges scores:

(2) \begin{equation*} S_j = \frac{1}{{\sigma _Z }}\frac{{\sum\nolimits_i {(Z_i - \beta _Z \mu _Z )} }}{{\sqrt M }} + W\frac{{(\sum\nolimits_h {E_h ) - \mu _{E(M)} } }}{{\sigma _{E(M)} }}. \end{equation*}

The first part is the same as Equation (1). The second uses a similar idea to score the edges in each sub-network. *E_i_* is the edge score defined for edge *i* for an edge in module *j, μ_E(M)_* and *σ_E(M)_* are, respectively, the mean and SD of edge scores calculated for a module of size *M*. Computing the background statistics for the edges score component is less straight forward as it is a function of the number of nodes. The number of nodes in a sub-network sets minimum and maximum limits on the number of edges in this sub-network. To learn the conditional distribution of edge scores (as a function of size, *μ_E(M)_, σ_E(M)_*) we used an iterative approach using randomized modules. We calculated mean and standard deviation of the edge score distribution over random modules for every possible module size *M* between 1 and 150. In addition, the score of the edge component in Equation (2) is dependent on the topology and density of the network. To balance the impact of edge component on the overall score of the sub-network, we combine the node score and edge score components using a tunable weight parameter *W*. This parameter, *W*, is a user-defined parameter controlling the weight of the edges score component w.r.t. the node component. Note that *W* differs from the confidence score given to each individual edge (*E_i_*). The optimal value of *W* is determined based on the parameter selection criterion described in the Section titled 'Parameter selection criterion' below. A higher *W* means more emphasis is placed on the connectivity.

### Searching for high scoring sub-networks

Greedy search was previously shown to produce good results when searching for sub-networks (16). Our search procedure starts by selecting seed nodes (e.g. highly activated nodes) that are expanded using breadth first search by evaluating the objective function described above. In each step, we add the node that maximizes the objective function for the subset of nodes that were previously selected. Nodes can be added to the component as long as the overall score of the component increases. We set an optional minimal active module size to five nodes which allows the algorithm to grow seeds with high initial scores. As nodes can appear in more than one module, highly intersecting modules are merged by keeping only edges that are appear in a high percentage of the modules. Edges that are found to be part of several modules starting from different seeds are more likely to be relevant to the analysis. This is evaluated by calculating the number of appearances of an edge *e(a,b)* between node *a* and node *b* in any module, divided by the maximum number of appearances of node *a* or node *b* in any module, and comparing this calculation to some user-defined cutoff. In set notation this can be written as the following.

For each $e(a,b) \in E$, $e(a,b)$ is kept if

(3) \begin{equation*} \frac{{\sum\limits_j {I(M_j ,a) \wedge I(M_j ,b)} }}{{\max (\sum\limits_j {I(M_j ,a)} ,\sum\limits_j {I(M_j ,b)} )}} {>} {\rm cutoff}, \end{equation*}

where *E* is the set of all edges, *e(a,b)* is an edge connecting nodes *a* and *b, M_j_* is a module identified by iterator *j* for all possible modules (*1,...,n*) and *I(M_j_,x)* is an indicator function of whether node *x* is part of module *M_j_*, for nodes *a* and *b*, respectively. A higher cutoff will only retain edges that have been frequently visited starting from different seeds nodes and would thus lead to smaller modules. However, varying the parameters within a reasonable range [0.1–0.4] had little effect on the assessment criteria (see the Parameter selection criterion and Supplementary Data Set DS2). Modules with less than four nodes were omitted.

### Parameter selection criterion

As with any data integration method, we need to determine the weight assigned to each data type (*W* in our model). The best method to determine parameter values is by using a gold standard set (e.g. known modules in the condition) and, in a training procedure, choosing values that lead to the best recovery of such known modules. However, in our case little is known about modules that are activated during different types of infection, and we expect this to be a general problem for other studies as well. Thus, to determine the value of this parameter we searched for values that optimize the following three general criteria (so that it is applicable across a wide range of conditions being studies): (i) the percentage of the number of modules that contain uniquely enriched Gene Ontology (GO) biological processes terms (i.e. terms that are found to be significantly enriched in only one module) (28). (Alternatively the KEGG biological pathways (29) can be used, although the granularity level of Kyoto Encyclopedia of Genes and Genomes (KEGG) is less refined compared to GO). This criterion examines the ability of the algorithm to capture distinct biological processes. (ii) The percentage of the number of DE nodes out of total nodes in the selected modules. This criterion aims to explain as many of the observed expression changes as possible. (iii) The total number of modules. This criterion attempts to balance the number of DE genes explained with the goal of selecting a set of distinct processes. Taken together the three criteria aim to produce the most relevant set of modules for the given expression data. Modules were tested on a variety of possible edge weight and beta parameter combinations and the values that maximize the above criteria are selected (see heatmap in Supplementary Data Set DS2).

### Network randomization

In order to evaluate the performance of ModuleBlast we generated random networks that preserve the degree distribution of the corresponding real networks. We tried two randomization methods: node expression value shuffling and edge switching. The first method is based on shuffling the expression values in each of the species, hence preserving the exact same topology of the original network. In the edge switching method we continuously picked two edges and switched their node assignments, hence preserving the degree distribution of each node (see the Supplementary Methods).

### Assessing module conservation

As mentioned above, for each node in our network we have at least two scores (one from each species). While we use the maximum absolute value when searching for sub-networks, once these are found, we can study and compare the activation of nodes from the two species in each module using their actual values. In order to evaluate the convergence or divergence of modules we calculated for each module the L1 distance over all the nodes in the module using the difference between the most extreme values in the genes represented by each node in the two species. Specifically, we compute

(4) \begin{equation*} {\rm Diff}(S_j ) = \frac{{\sum\nolimits_i {\left| {Z_{i\,A} - Z_{i\,B} } \right|} }}{{\sqrt M }}, \end{equation*}

where *S_j_* is a sub-network of size *M, Z_iA_* and *Z_iB_* are node scores from species *A* and *B*, respectively. The distance obtained for each module was compared to distances calculated for 10 000 random modules with the same number of nodes, using nodes that are part of some module. In addition, we assessed the overall activation/repression of the modules in each of the species using a similar randomization method over the sum of values in nodes for each of the species separately, i.e.

(5) \begin{equation*} {\rm Active}(S_j ,X) = \frac{{\sum\nolimits_i {\left| {Z_{i\,X}} \right|} }}{{\sqrt M }}, \end{equation*}

where *S_j_* is a sub-network of size *M* and *Z_iX_* is a node score for all nodes *i* in *S_j_* from species *X*. Similar to the Diff calculations, we compared the obtained distances to distances calculated for 10 000 random modules with the same number of nodes, using nodes that are part of some module.

Using these measurements we classified the modules into three categories: (i) conserved modules. These modules show little difference between the species compared to random modules (Diff*(S_j_)* > 0.95), i.e. at least 95% of the random modules are more divergent than the inspected module. (ii) Modules that are species specific. Modules that are different between the species (Diff*(S_j_)* < 0.05) and show high activation (Active*(S_j_,A)* < 0.05) in one of the species and low activation in the other (Active*(S_j_,B)* < 0.05). (iii) Divergent modules that are divergent in opposing patterns (e.g. one is upregulated and the other is downregulated). These modules are highly different between the species (Diff*(S_j_)* < 0.05) and show high activation in both species, i.e. Active*(S_j_,A)* < 0.05 and Active*(S_j_,B)* < 0.05). Note that several modules fall outside all three categories (divergence score between 0.05 and 0.95). While such modules are still very relevant to the condition being studied, for these modules we do not make a call regarding conservation.

### Matching modules through time

In order to identify cascades of activated modules, we generated a separate modules set for each time point using a search procedure that is similar to the one described above. We next tested the overlap of module sets between time points using a hypergeometric test. If reciprocal tests were found to be significant (*P*-value < 0.01), we defined these modules as matching. In many cases clear chains are identified throughout the time series indicating a module that is preserved through time. Nonetheless, usually in earlier time points where the overall activation of modules is lower there may be several modules that are matched to later time points creating a fan-in structure.

### Enrichment analysis

Enrichment analysis for each module was performed using GO biological processes (28), KEGG pathways (29) and all possible sets defined in GSEA (30) (that include gene sets from KEGG, REACTOME (31) and BIOCARTA). Transcription factors regulating modules were inferred using data from TRANSFAC (32). Annotation information for mouse and human was combined. Multiple hypothesis testing corrections were performed using Bonferroni correction for GO, KEGG, GSEA enrichment and FDR for TF enrichment. Full analyses are available in Supplementary Data Sets DS4–DS7 correspondingly.

## RESULTS

### Cross species interaction networks

Interaction networks that combine information from both species being compared are assembled as discussed in the Materials and Methods section using available genomic data from the two species. Once we have networks that integrate information across species, we use a novel target function that takes into account both activity and connectivity to search for active sub-networks. Our target function is useful for identifying not just the active/repressed genes but may also identify genes that are post-transcriptionally regulated being impacted by the expression of their neighbors in the network. We use a greedy search method for finding modules (sub-networks) that maximize the target function. Highly overlapping sub-networks are merged based on the quality of the overlap (the Materials and Methods section). These networks can be used to identify both similarities and differences between species. In addition, the modules can be connected over time when using time series expression data to identify the progression of information within cells. A general outline of our methods for assembling and searching the combined interaction network is presented in Supplementary Figure S1. Simulation result indicate that ModuleBlast is able to identify conserved and species specific activated modules for the vast majority of tested cases. More importantly, the method correctly assigns the ‘conserved’ or ‘divergent’ label for almost all identified modules indicating that such assignments are robust; see the Supplementary Results for details.

### Comparing mice and macaques

We next applied ModuleBlast to study the response of alveolar macrophages (AM) from mice and cynomolgus macaques to *F. tularensis* Schu S4. *F. tularensis* causes a wide range of infections, including pneumonias of the lower respiratory tract. In order to understand how prevalent these cases are, we calculated statistics for the number of members in each orthogroups between mouse and macaque. 80.1% (11 368 out of 14 200 orthogroups) have only two members corresponding to a 1:1 orthology match (See Supplementary Data Set DS1). 16.5% (2337) have three members (corresponding to 1:2 or 2:1 matches). Only 0.3% of the orthogroups have six members or more, indicating that paralogs are not likely to cause a large shift in the results. This was also confirmed by repeating the analysis using the mean expression value over the group of paralogs instead of using the most extreme value, which resulted in nearly identical modules.

### Functional analysis of modules across species

Using ModuleBlast we obtained 17 modules containing 188 unique nodes (Supplementary Data Set DS3), out of which 13 modules were enriched for unique GO terms. The ability of the method to identify significantly enriched modules with relevant functions while at the same time minimize overlap between these modules highlights the ability of ModuleBlast to identify distinct mechanisms triggered by the infection. 43 unique GO terms, 36 unique KEGG pathways and 154 GSEA sets were identified for all modules (Supplementary Data Sets DS4, DS5 and DS6), including modules that are enriched for chemotaxis, transfer of antigenic peptides (TAP) complex, apoptosis and NFκB regulation, all relevant to the strategies employed by *F. tularensis* upon infection of the cell (see further discussion below). Of the 188 nodes, 52.13% show a high differential expression in either of the species. The modules contain 602 unique edges (3.2 edges to nodes ratio, 0.0343 graph density) indicating a high connectivity in the resulting modules.

We conducted several tests to evaluate whether cross species analysis improves our ability to identify relevant modules. We first tested the ability of our method to capture insights across species by comparing the combined mouse–macaque analysis with analyses that were conducted separately for each of the species using species-specific expression data and complete interaction networks (including genes that do not have orthologs in the other species). As can be seen in Table 1, cross species analysis leads to more modules, a larger number of nodes and a significantly larger number of unique enriched GO terms and KEGG pathways. Importantly, none of the enriched modules that were identified using the individual species data are enriched with the TAP complex or the apoptotic processes which play significant role in the *F. tularensis* infection (see below). Supplementary Data Set DS4 marks GO categories that are unique to the joint analysis (species-specific GO analysis is available in Supplementary Data Sets DS8 and DS9). We next compared our results to analyses that are based on randomization tests. We used two variants for the tests: expression value randomization (that keeps the exact same network topology as the real data) (Supplementary Table S2), and edges switching (that keeps the same expression for nodes but changes the network topology) (Supplementary Table S3). See the Materials and Methods section and Supplementary Methods for details. In both cases, the number of modules as well as the number of nodes identified by ModuleBlast significantly decreased. Both of these randomization methods retain the underlying network distributions.

**Table 1.** Comparing ModuleBlast to species specific, random and other methods

	ModuleBlast	Species specific		Cross species programs		Single species programs			Expression only
	Joint species analysis	Mouse only	Macaque only	NeXus*-*conserved	Nexus*-*sp specific	jActiveModules	COSSY	GXNA	*K*-means
# of modules	17	8	14	907	1888	50	8	25	100
% of uq nodes	**100%** (188/188)	**100%** (76/76)	**100%** (105/105)	0.50% (1749/386851)	0.10% ^(2838/2403084)^	11.50% (1694/14722)	90% (36/40)	38.70% (145/375)	**100%** (14200/14200)
% of uq active nodes	**52.10%** (98/188)	31.60% (24/76)	31.40% (33/105)	35.6% (623/1749)	18.10% (514/2838)	40.9% (692/1694)	27.80% _(10/36)_	20% (29/145)	15.50% (2203/14200)
% uq enriched GO modules	**76.50%** (13/17)	50% (4/8)	28.60% (5/14)	3.6% (33/907)	1.60% (30/1888)	34% (17/50)	50% (4/8)	0	2% (2/100)
% uq enriched KEGG modules	47% (8/17)	37.50% (3/8)	7.10% (1/8)	0.70% (6/907)	0.20% (3/1888)	12% (6/50)	**75%** (6/8)	0	0% (0/100)
% uq enriched GSEA modules	76.50% (13/17)	50% (4/8)	0% (0/8)	6% (54/907)	2.60% ^(49/1888)^	80% (40/50)	**87.50%** (7/8)	0	2% (2/100)
# of uq GO terms	43	28	5	158	243	77	13	0	5
# of uq KEGG pathways	36	5	1	62	67	37	11	0	0
# of uq GSEA sets	154	29	0	598	815	437	27	0	4
Running time	**s**	**s**	**s**	15 h	79.2 h	3 m	**s**	**s**	**s**

ModuleBlast results using joint mouse and macaque data (first column) compared to analyses using only mouse or only macaque expression data and network (second and third columns), and several other sub-network search methods including two modes of operation of NeXus (conserved and species specific), GXNA, COSSY and jActiveModules. The last column shows clustering expression values with no network information. Rows definitions: the number of modules, percent of unique (uq) nodes out of the nodes in the analysis, percent of unique active (DE) nodes (scaled fold change > 0.25), percent of modules that have at least one uniquely (uq) enriched GO term, KEGG pathway or GSEA set, the total number of unique (uq) GO terms, KEGG pathways or GSEA sets. The best values in percentage categories are marked in bold. For running times, **s** - seconds, m - minutes, h - hours.

### Comparing ModuleBlast to other methods

While several methods were developed for finding active sub-networks in a single species, as mentioned in the Introduction section, only one previous method (NeXus (22)) has been developed for cross species identification of such modules. In addition, several of the single species based methods discussed above do not provide an implementation making it hard to directly compare our method with these previous methods (in this setting, once the networks are formulated, a single species method can be used as well). We have thus compared our results with NeXus (22) as well as multiple single species algorithms for which we could find a standalone implementations including jActiveModules (1), GXNA (16) and COSSY (33). MATISSE (34) was not able to scale to the size of our network. In addition, to test the usefulness of the network information we have also compared to a simple clustering method (k-means) that only uses the expression values of the set of orthologous genes. The results are summarized in Table 1. See comparison details in Supplementary Methods. Our results are compared using GO annotations as well as KEGG pathways (29) and GSEA terms (30) (both were not used in our target function). Because of comparison reasons with NeXus, node and edge scores were predetermined based on the orthology information and scaled to [−1,1] range. In most comparisons, ModuleBlast identified the largest percentage of unique active nodes out of the nodes that are part of the analysis (52.13%) as well as the largest percentage of uniquely enriched modules using either biological ontology (e.g. 76.47% GO enrichment). NeXus, the other cross-species module search program, has two distinct module search modes: conservation and species-specific. Unlike ModuleBlast which searches for conserved and divergent modules at once, NeXus requires two independent runs which result in two distinct module sets, hence the NeXus results are reported separately for each mode. As can be seen, for these data NeXus identifies significantly more modules than ModuleBlast in both modes (17 versus 907/1888). However, many of these extra modules are highly overlapping and 99% of the nodes appear in more than one module. This may present a problem when searching for few distinct modules for follow-up experimental analysis. Indeed, while the number of modules obtained by NeXus is 164 times (combined) the number of modules identified by ModuleBlast, only a small percentage of them is uniquely enriched with specific GO categories (76.47% versus 3.64%/1.59%). Modules that were obtained in NeXus's species-specific mode and in the conserved mode were found to be highly overlapping and could not easily classify biological functions as species-specific or conserved. Another problem when using NeXus is its runtime. NeXus required almost 4 days to run (combined) whereas ModuleBlast terminates in few seconds on this data set (all tests were performed on a standard dual core desktop workstation). In addition, we compared the ability of ModuleBlast and NeXus to obtain relevant modules compared to random by shuffling expression values (Supplementary Table S2). Due to the long running time of NeXus only one random analysis was performed for NeXus. ModuleBlast found 41% more modules for the real data compared to random, while NeXus found only 10% more in the conserved setting and about the same for the species-specific setting. In terms of the number of uniquely enriched modules (GO/KEGG) ModuleBlast had a 38% improvement compared to random while for NeXus the percentages where comparable between the real and random data (Supplementary Table S2). jActiveModules is a single species module search that is provided as a plugin for *Cytoscape* (35). jActiveModules, requires the user to provide the number of modules to look for. Using the default settings which searches for 50 modules and allows at most 80% overlap between modules (Table 1), most of the modules were found to be highly overlapping, quite large and less indicative for specific roles modules may be involved in. In another setting, in which no overlap was permitted, only one large module containing more than 400 nodes was identified by jActiveModules (not shown). GXNA, another single species program, also requires the user to define the number of modules and their maximum size. Using its default settings, GXNA generated 25 modules each containing 15 genes. However these modules were highly overlapping and were not found to be enriched with known biological functions thus difficult to use for practical follow-up analysis. COSSY is also a single species analysis method. For this comparison we treated the two species as two conditions and mapped all the values to the mouse gene names. As can be seen in Table 1, while the result from COSSY appear to be better than the other methods we compared to (finding a small set of non-overlapping modules, several of which were enriched with GO and KEGG terms), the total number of modules COSSY identified for these data and the number of genes in these modules were very small (8 and 40, respectfully). In contrast ModuleBlast identified more than twice the number of modules and four times the number of genes leading to a much higher set of identified functional and KEGG categories. Clustering the gene expression data using *K*-means (*K* = 100) was not able to find clusters associated with relevant biological functions since, unlike the other methods, it does not use the network information that may have made it hard to focus on a subset of the genes.

### Evaluating divergence and conservation

**Figure 1.**
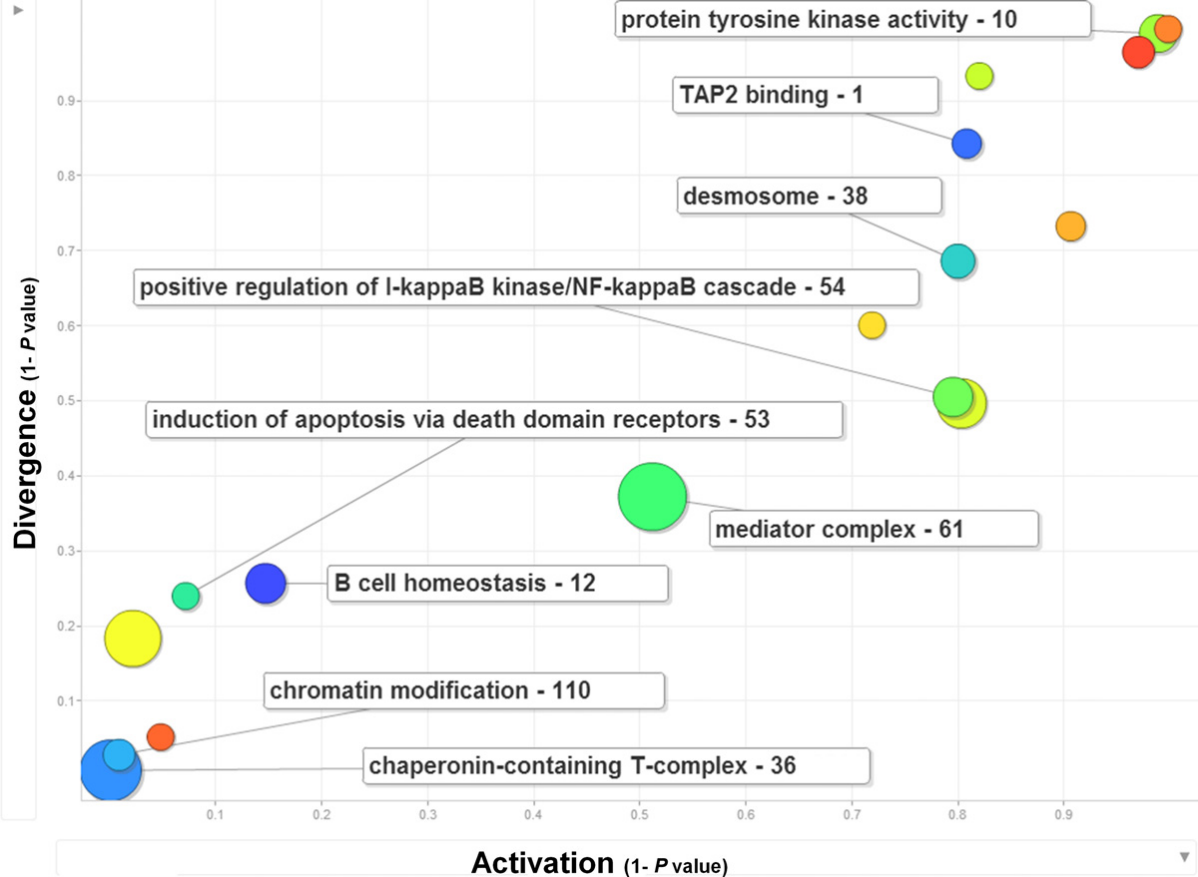
Activation and divergence of key modules. The activation measure is plotted against the divergence measure for several selected modules. The most significant GO biological process term is shown for selected modules. The number near each module represents the module ID. Modules on the top are more divergent (1—Divergence *P*-value as defined in equation (4)). Modules on the right show higher activation in either of the species (1—Activation *P*-value as defined in equation (5), but calculated on both species together, leading to the linear shape of the scatter plot). The size of each node is proportional to the number of genes in the module.

We next assessed the modules to determine if they are conserved or divergent across the two species. Conservation is multifaceted when examining modules across species. The three options for conservation and divergence we considered are: (i) conserved modules (CM), (ii) divergent modules that are species-specific (SP), i.e. active in only one of the species and (iii) divergent modules that show opposite expression patterns in the two species (OP) (the Materials and Methods section). In order to evaluate the convergence or divergence of the modules we calculated the sum of differences between the various species over all the nodes in the module and compared these differences to 10 000 random modules with the same number of nodes (the Materials and Methods section). Conservation and divergence are determined by looking at the absolute difference between expression values for nodes from both species. This may bias the analysis in cases of correlated expression changes leading the method to miss conserved modules. In addition, variance in module node values will affect the assignment to conserved/divergent categories, as our method does not assume that divergent modules should have a consistent difference (e.g., one high and one low) between the two species. We also assessed the overall activation of the modules in each of the species using randomization methods. Out of the 17 modules, we classified four modules as conserved (CM), two modules as species specific (SP), and two module as divergent (OP) using the cutoffs defined in the Materials and Methods section (Supplementary Data Set DS4). Comparing modules overall activation and divergence revealed high correlation between them. Figure 1 plots overall activation versus divergence for some modules with significant GO enrichment (the most significant GO term is shown for each module). Note that in this figure the activation axis combines information from both species together, leading to the highly linear shape of the scatter plot. GO enrichment analysis of the conserved modules was found to be related to several biological processes including chromatin modification and chaperonin containing T-complex, a process which was shown to play role in ciliogenesis, specifically in airway infections (36).

**Figure 2.**
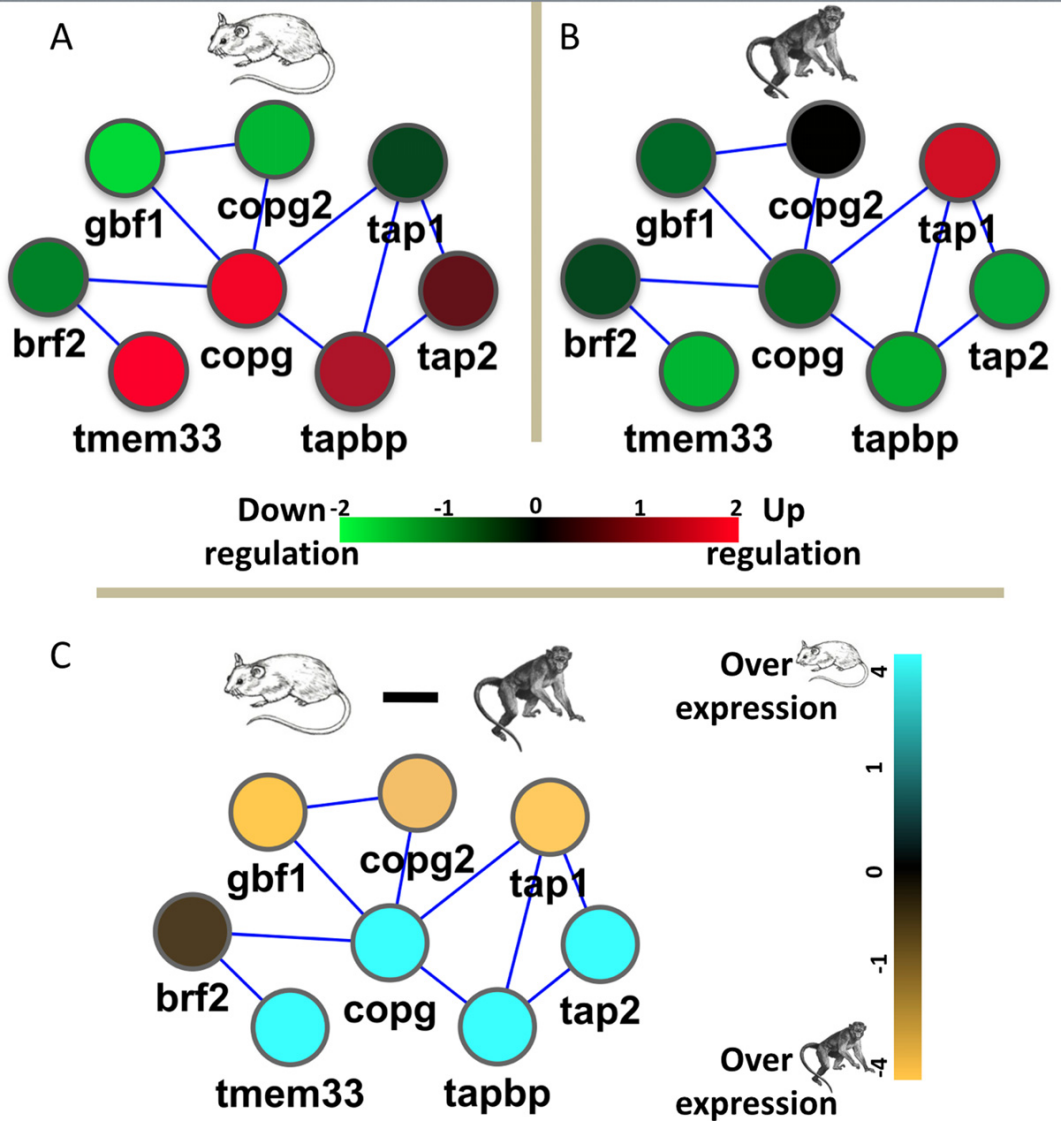
Differences between species. Module 1 is one of the divergent modules in opposing directions (OP) and involves the transfer of antigenic peptides (TAP) complex, which was previously shown to be transcriptionally active after *F. tularensis* infection in human cells. (**A**) Mouse expression values. (**B**) Macaque expression values. (**C**) Macaque expression values subtracted from the mouse expression values, highlighting similarities and differences between the species. Note that the species-difference scale is set to a minimum and maximum of 4-fold change compared to a 2-fold change for each species separately. Mouse gene names are used.

The divergent modules highlight processes that may respond differently to *F. tularensis* infection. For example, the mouse-specific module 10 was found to be enriched with KEGG pathway of chemokine signaling pathway and GO process of serine/threonine kinase activity that was shown to be involved with *F. tularensis* infection (37). In another example, one of the relatively divergently opposite modules, module 1, was enriched for TAP complex and in KEGG pathway for Antigen processing and presentation (Figure 2). This complex is known to be involved in the transport of antigens from the cytoplasm to the Endoplasmic Reticulum (ER) for association with the major histocompatibility complex (MHC) class I molecules and TAP1 was previously shown to be transcriptionally active after *F. tularensis* infection of human cells (38–40) and was found to be one of the genes that could best separate tularemia patients from convalescent patients (38).

### *F. tularensis* induces changes in apoptotic expression

Module 54 (Supplementary Figure S2), is relatively divergent across both species and is highly enriched for apoptosis (1E-6) and positive NFκB regulation (1E-6). Apoptosis was previously shown to play an important role in murine *F. tularensis* infection, killing macrophage cells in 24–48 h (41). TF enrichment analysis for this module found RelA-p65, an active form of NFκB to be the most enriched TF regulating this module (1E-3; see Supplementary Data Set DS6). Aberrant NFκB activation by *F. tularensis* coupled with differential activation of NFκB in murine versus primate cells could contribute to the differences observed in Supplementary Figure S2 and influence apoptosis-related genes. In support of this, Module 54 contains transcript for a number of pro- and anti-apoptotic molecules including TNF, TRAF1, TRAF2 and TRADD. All of these show significant expression changes between 2 and 6 h after *F. tularensis* infection in both species. NFκB is regulated by the heterodimeric TRAF1/2 complex that interacts with the inhibitor-of-apoptosis proteins and TRADD to mediate an anti-apoptotic signal from the TNF receptors. Independent evidence also supports this interrelationship; a related bacterium, *Francisella novicida*, was recently shown to block staurosporine-induced apoptosis in macrophages, which correlated with activation of nuclear transcription factor B (NFκB) (42).

### Response progression over time

The above analysis was conducted by averaging the entire time series values for each gene into a single value. While useful for finding relevant functional modules, we sought to identify the entire cascade of events that occur following *F. tularensis* infection over time. We therefore constructed a module set for each time point separately and matched the resulting module sets in each time point to all other time points using a reciprocal hypergeometric test (see the Materials and Methods section). General trends through the time course analysis show that the size of the modules and the number of enriched GO terms significantly increase as the response progresses.

**Figure 3.**
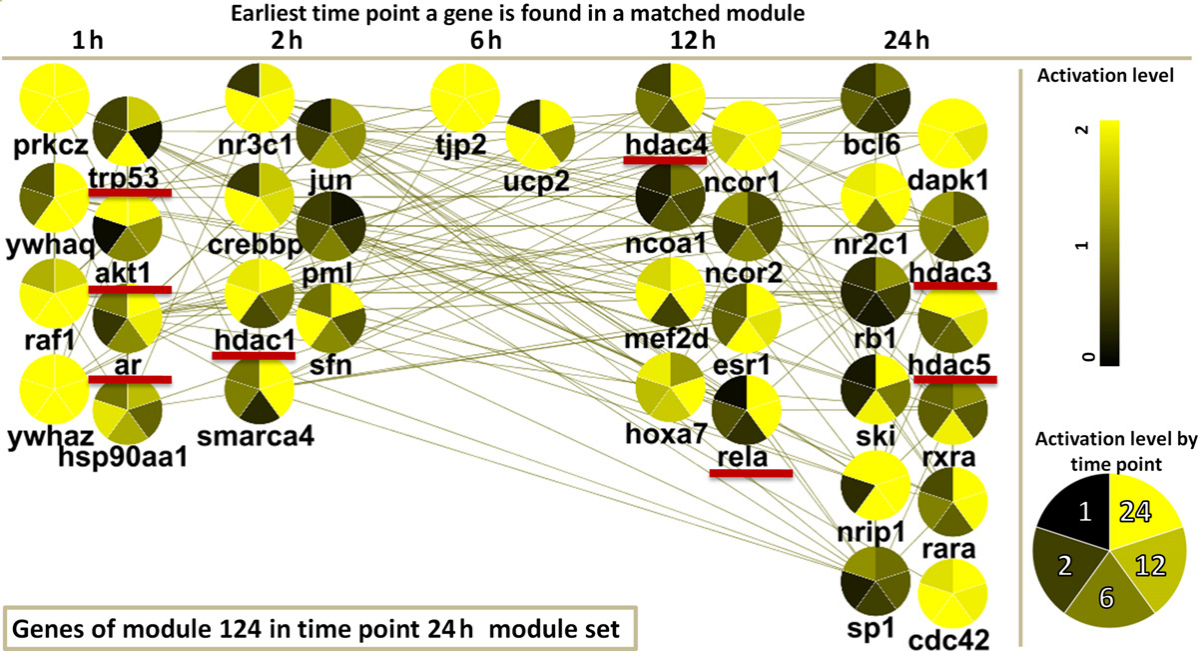
Response progression over time. Modules were created for each time point and matched using reciprocal hypergeometric test. Module 124 in time point 24 h is shown in a layout that depicts the module expansion through time; genes that are part of matched modules in earlier time points are placed in columns from left to right based on the earliest time point they were found to be part of a matched module. The number of nodes and their activation level increase over time. Node coloring is based on overall activation (up or downregulation) of the orthogroups in 1–24 h. This module is enriched with regulatory factors, including TP53, relA, AR and AKT1 that are involved with the *F. tularensis* infection (see text).

In one example, module 124, in time point 24 h is enriched with transcription regulation and is reciprocally significantly matched to modules in all earlier time points (the Materials and Methods section). Figure 3 plots the genes in module 124 in five columns based on the earliest time point a gene was part of a matched module. Each node is colored by the expression level at all time points in a counter-clockwise fashion. It is easy to see that in this module the number of genes and the overall activation of the genes (up or downregulation) increased over time. Module 124 is enriched with important transcription regulators, including p53, Jun, RelA (NFκB p65), CREB binding protein and histone deacetylases (HDAC) 1, 3, 4 and 5. TP53 plays role in apoptotic and anti-apoptotic processes, and its expression was increased in 6 h. RelA, a part of the NFκB complex, increased at 12 h and is a pro-inflammatory transcription factor that also triggers anti-apoptotic responses. HDACs, which are shown to be transcriptionally active in late time points can regulate the function of NFκB and TP53 (43,44). Pro and anti-apoptotic processes may play a significant role during *F. tularensis* infection (see above). Module 124 also contains several genes that were found in matched modules in early time points. Specifically, AKT1 expression is elevated 1 h after exposure to bacteria, during the *F. tularensis* penetration, but not in later time points. This result is similar to previous observations that *F. tularensis* Schu S4 infection reduces AKT1 gene and protein expression, thereby reducing cytokine response and host defenses against infection (45) Another gene that is active in early time points is androgen receptor (AR), a nuclear receptor that is regulated by target lesson revascularization stimulation and interferon-γ in macrophages (46). In addition, we found immune modules enriched with chemotaxis, cytokine activity and chemokine activity. Specifically, for module 34 in the 24-h module set, we observed early activation of chemokine ligands CCL20 and CCL8. CCL8 interacts with CCR10, a chemokine receptor that is activated at later time points when both are assigned to the same module. Taken together, these results indicate that the temporal matching method can identify relevant associations when integrating expression and interaction data.

### Cross species analysis of aging

The analysis of immune response data described above allowed us to compare ModuleBlast to other methods based on curated sets of immune response genes. However, ModuleBlast can also be used in cases where little prior biological knowledge is available. To illustrate this and to test the ability of such an analysis to generate new testable hypotheses, we applied ModuleBlast to study data regarding the SIRT6 (sirtuin 6) protein. This protein was implicated in calorie restriction response (47) and its deficiency in mice leads to premature aging and metabolic defects, resulting in premature death (48). In a recent study we showed that over-expression of SIRT6 extends lifespan of male mice (49), and protects against high-fat-diet-induced obesity, although we still do not fully understand the exact mechanism by which SIRT6 operates.

To study the mechanisms by which SIRT6 affects aging and to determine the relationship between mice and human responses we used ModuleBlast to compare expression data sets from mouse embryonic fibroblast cells (MEF) extracted from a knockout SIRT6 mouse (KO mice) and a human HeLa cell line expressing shRNA against SIRT6 (KD human) (26). We found that in the absence of SIRT6, the resulting modules for the mouse–human comparison are enriched with chaperonin containing T-complex, ubiquitination, DNA binding, RNA splicing and positive regulation of NFκB. Importantly, we found a significantly conserved immune and inflammatory response module in which some of the genes downregulated in both human and mouse while others, including TNFR2 in the human module and TRAF in the mouse module, were upregulated (Supplementary Figure S3).

### Experimental validation of hypotheses resulting from our analysis of the ModuleBlast results

**Figure. 4.**
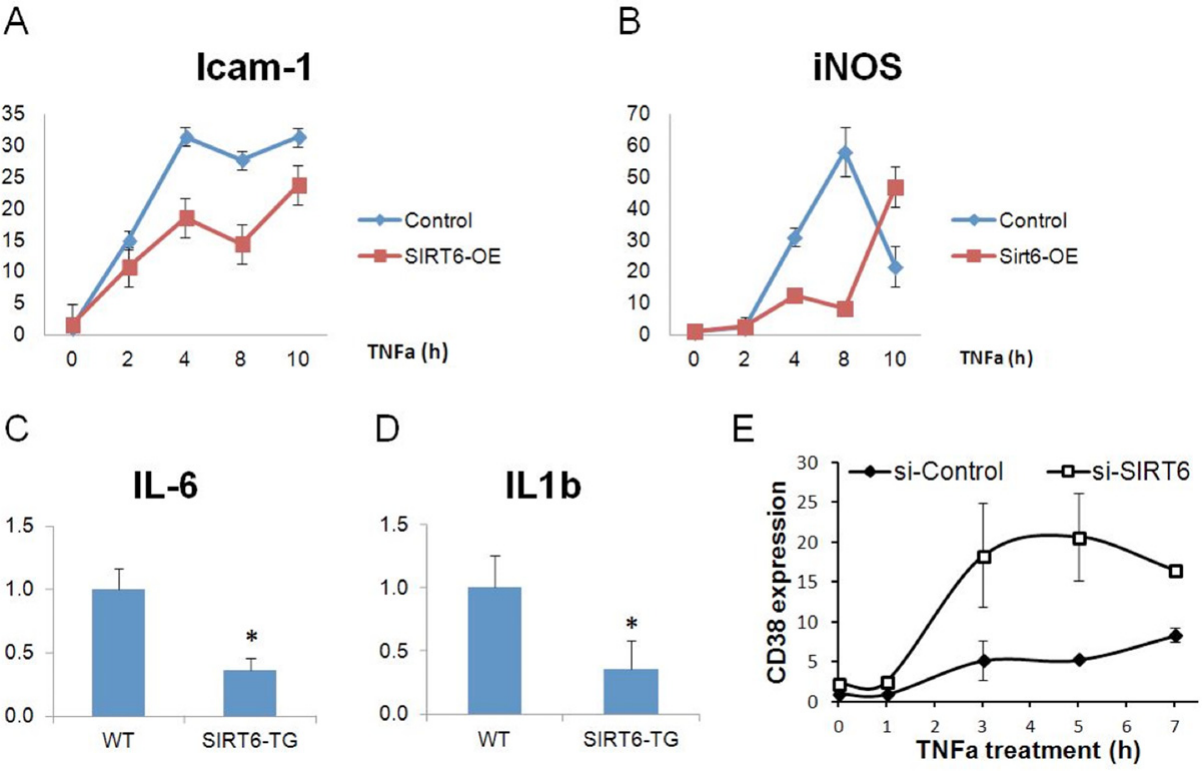
Follow-up analysis of Sirt6 regulation. *In vitro* mRNA levels of ICAM-1 (**A**) and iNOS (**B**) in 3t3-L1 cells stimulated with TNF-α. (**C**) *In vivo* IL-6 mRNA levels in SIRT6-overexpressing mice fed high fat diet (HFD) (**D**) IL1β mRNA levels in SIRT6-overexpressing mice fed HFD, *n* = 7 or 8 per group. (*P < 0.05 based on a 2-tailed *t*-test). (**E**) *In vitro* mRNA levels of CD38 in HeLa cells stimulated with TNF-α.

The role SIRT6 plays in controlling inflammation has been somewhat controversial. On the one hand, the study that generated the KO and KD expression data (26) indicated that SIRT6 may have a protective anti-inflammatory role, as lack of SIRT6 causes increased inflammation. However, a study using human HEK293 kidney cells did not find such protective role for SIRT6 over-expression 1 h after treatment with the tumor necrosis factor (TNF-α), an inflammatory agent (50). ModuleBlast analysis of KO and KD SIRT6 in human and mouse cells identified modules containing important immune response regulators, including the activation of TNFR2, a member of the tumor necrosis factor receptor family. We thus decided to experimentally test the effect of modulation of SIRT6 levels on classical markers of inflammation, both in vitro and in vivo in both human and mouse by treating cells with TNF-α (which acts through TNFR2) over a 10-h period (as opposed to only 1 h tested by (50)). Pre-adipocyte 3T3-L1 mouse cells stably over-expressing SIRT6 were stimulated with TNF-α, and select inflammatory genes were examined. As seen in Figure 4A and B, following treatment with TNF-α, after 2–4 h SIRT6 was able to inhibit the increased response of the classic inflammatory gene ICAM-1 and delay the inflammatory response of iNOS, demonstrating that over-expression of SIRT6 protects against these TNF-α-mediated inflammatory genes. Following our in vitro validation, mRNA levels of adipokine gene IL-6 were measured in SIRT6 over-expression mice during high fat diet (HFD), a diet known to increase inflammation. IL-6 is a classic pro-inflammatory adipokine secreted from the white adipose tissue (WAT), and mice fed HFD have increased levels of this adipokine. We found a significant decrease in the average levels of IL-6 mRNA in WAT from mice over-expressing SIRT6 (Figure 4C). Additionally, we examined the transcription levels of the inflammatory gene IL1β in mice fed HFD and found that SIRT6 over-expression significantly decreased levels of IL1β as well (Figure 4D). These results indicate that SIRT6 may protect against inflammatory cytokines produced by the WAT tissue in HFD mice.

We have also repeated these experiments in a human cell line (HeLa cervical cancer cells), to confirm that the immune response is decreased in human cells as well. Downregulating SIRT6 in TNF-α-stimulated HeLa cells results in significantly increased levels of the inflammatory gene CD38 (Figure 4E). Taken together, these experiments support the hypotheses derived from the ModuleBlast results indicating that SIRT6 plays an important role in regulating inflammatory response starting at 2–4 h after TNF-α treatment via regulation of the TNF module.

## DISCUSSION

We developed a novel method to find active sub-networks within and across species. ModuleBlast provides enhanced cross species capabilities by classifying the modules to several conservation types and identifying modules that show interesting activation patterns. Identifying conserved and divergent response patterns in the context of connected groups of genes is becoming increasingly important with applications for both basic science and drug discovery research by highlighting biological mechanisms that are likely to be affected similarly or differently to a specific drug or treatment. We applied ModuleBlast to a time series of expression data from mouse and macaque AMs infected with *F. tularensis* and found several modules with high relevance to the response progression over time and immune response mechanisms. The combined species analysis was able to identify modules that were not found in the single species analyses and show improved statistics over other methods. We have also applied it to study the effects of one of the first validated mammalian aging proteins, SIRT6. Follow-up experimental analysis, both in vitro and in vivo, confirmed the inflammatory response role for SIRT6 that was predicted by ModuleBlast.

It should be noted that ModuleBlast is intended to serve as a discovery platform and not as a detailed mechanistic modeling framework for gene regulation. An underlying assumption in our analysis is that paralogous genes are likely to show similar expression patterns and their measurements can be summarized by taking the most extreme value for the maximum possible activation for all paralogs. This assumption is not always realistic and paralogous genes may exhibit quite distinct behavior. In addition, no ‘gold standard’ or large-scale experimentally validated information is currently available in order to validate the resulting modules. We have thus used GO, KEGG, GSEA and follow-up experiments to determine functional enrichment and validate some of our modules. While such an analysis cannot serve a conclusive evidence for the accuracy of the modules, the increase in the number of observed terms from all three annotation sets when compared to the randomized results, and the agreement between the predictions and experimental results, supports the modules identified by ModuleBlast.

A related prior work is the ‘phenologs’ approach by McGary *et al.* (51) which looks for phenotypes across species that intersect by a large fraction of orthologous genes. Whereas ModuleBlast attempts to determine gene function and cross species relationships by relying on new quantitative experimental data, the ‘phenologs’ work requires known assignments of genes to phenotypes in each species and is thus not intended to the same type of studies that ModuleBlast is intended for.

ModuleBlast constructs a joint network for both specific being studied. An interesting future direction would be to develop similar methods that can perform cross species module analysis without the need to (artificially) combine the two networks. Such an approach would need to rely, at least in part, on network alignment methods and these can be computationally challenging though several heuristics for such problems have been suggested (52).

We have implemented ModuleBlast as a general purpose, easy-to-use web tool with built-in support for various gene identifier names spaces, orthology information and underlying networks (See Supplementary Figure S4). The web tool can be used at the URL provided in the Abstract page, requires only gene identifiers and values to operate and offers extensive analysis options of the results. We hope that our tool will be a useful addition to the current set of analysis packages used by the experimental and computational communities.

## AVAILABILITY

Supporting web server: www.expression.cs.cmu.edu/module.html

## SUPPLEMENTARY DATA

Supplementary Data are available at NAR Online.
